# Examining the Relationship Between Grit, Passion, and Professional Attitudes in Nursing Students: A Cross‐Sectional Study

**DOI:** 10.1111/scs.70079

**Published:** 2025-07-02

**Authors:** Seçil Taylan, İlknur Özkan

**Affiliations:** ^1^ Surgical Nursing Department, Kumluca Faculty of Health Sciences Akdeniz University Antalya Turkey; ^2^ İnternal Medicine Nursing Department, Kumluca Faculty of Health Sciences Akdeniz University Antalya Turkey

**Keywords:** grit, passion, professional attitudes, professional behaviours, quantitative

## Abstract

**Background:**

Grit, characterised by perseverance and passion for long‐term goals, is essential in achieving personal and professional success. This study aims to examine the relationship between grit, passion, and professional attitudes and behaviours in nursing students, drawing on grit theory.

**Method:**

A descriptive relational study was conducted with 207 nursing students. Data were collected using the ‘Passion Scale’, ‘Grit‐S Scale’, ‘Nursing Students Professional Behaviours Scale’, and the ‘Professional Attitude Tool for Student Nurses’. Multiple linear regression was used for analysis.

**Results:**

A positive correlation was found between students' professional attitudes and behaviours, and their levels of passion and grit (*p* < 0.001). Increases in Grit and Passion scores were associated with corresponding increases in Professional Attitude and Behaviour scores.

**Conclusion:**

The results suggest that personal traits such as grit and passion significantly influence nursing students' professional development. Incorporating strategies to enhance these traits may improve nursing education outcomes.

## 1 Introduction

Grit has been identified as an important personality trait in achieving goals. The personality trait of grit, defined as “perseverance and passion for long‐term goals”, is associated with goal achievement and performance outcomes. Passion and grit are critical elements that significantly impact the educational experience of students and shape their success [1, 2, 3]. While passion reflects an individual's will and energy to pursue an activity, grit refers to the psychological resilience and optimism displayed in the face of difficulty. Studies show that passion creates lasting motivation in learning and inspires students to become experts in a field. On the other hand, grit is cited as the key to achieving long‐term academic goals and maximising individual potential [4, 5, 6].

Passion and grit have become the focus of educational research. However, there is limited research on nursing education [7, 8, 9]. These studies have shown that grit and passion are important determinants of clinical and academic performance in nursing students [7, 8, 9]. Nursing education is not limited to clinical skills and theoretical knowledge, but also focuses on helping nursing students develop professional attitudes and behaviours [10, 11]. The concept of professionalism means that the internal values of the individual, such as conscience, honesty, and respect, as well as the elements required for professional identity, such as knowledge, autonomy, awareness, voluntariness, and teamwork, come together and are expressed in personal behaviour. The professional attitudes and behaviours that nursing students acquire during their education play a vital role in the effectiveness and quality of health services. In order for students to be able to transfer professional attitudes and behaviours to the clinical setting, it is important to provide appropriate opportunities for the formation and development of perceptions of professionalism during the educational process and for this to be reflected in patient care [10, 12]. In this process, identifying the factors that influence professionalism can guide the design of nursing students' educational experience. In this process, the educational life of nursing students can be guided by identifying the factors that influence professionalism [13, 14, 15]. However, no study was found that examined the relationship between passion and grit and the professional attitudes and behaviours of nursing students.

## 2 Theoritical Framework

Duckworth's grit theory was proposed in 2007. It states that perseverance and passion are fundamental to achieving goals. Grit as a personality trait goes beyond resilience and conscientiousness; it is characterised by perseverance and passion [6, 9, 16]. Duckworth et al. [6] highlight the interrelated dimensions of grit. These include persistence of effort and consistency of interest (passion). Perseverance of effort reflects the determination to persevere in the face of challenges, while Consistency of interest reflects a sustained commitment and enthusiasm for long‐term goals [6]. Duckworth's theory emphasises the essential role of passion, particularly in maintaining a consistent interest in pursuing goals. The passion component of grit is crucial in predicting performance [5, 17]. This emphasis on passion distinguishes the construct of grit from other predictors of success, such as perseverance, self‐efficacy and hope. Duckworth's grit theory emphasises that determination and passion are essential to achieving goals. Based on grit theory, this study hypothesises that professional attitudes and behaviours will be positively influenced by increasing passion and grit levels in nursing students.

## 3 Method

### 3.1 Type of Study

It is a descriptive, relational study.

### 3.2 Participants and Sample

The research was conducted in the Fall semester of the 2022–2023 academic year, between 10 and 29 October 2022, at the Faculty of Health Sciences of a state university in the Mediterranean region. The study population consisted of 216 students in their second, third, and fourth year of nursing education who had spent at least one semester in clinical practice. There was no sampling in the study, and the number of students who volunteered to participate in the study was calculated to be 139 students with a 5% confidence interval using the convenience sampling method. 207 students took part in the study. 95.8% of the population was reached.

### 3.3 Data Collection Tools

Research data were collected with the “Introductory Information Form”, “Passion Scale”, “Grit‐S Scale”, “Nursing Students Professional Behaviors Scale” and “Professional Attitude Tool for Student Nurses”.

#### 3.3.1 Socio‐Demographic Characteristics Form

The form prepared by the researchers includes questions about students' demographic characteristics, such as age, gender, year of study (class), economic status, reason for choosing the profession, and post‐graduation goals.

#### 3.3.2 Nursing Students Professional Behaviours Scale (NSPBS)

NSPBS; developed by Göz and Geçkil in 2010 to assess the professional behaviour of nursing students. This scale consists of a total of 27 items and is a five‐point Likert scale with three subscales: health care practices (18 items), activity practices (seven items) and reporting (two items). The total score that can be obtained from the NSPBS scale varies between 27 and 135. A high score on the scale indicates that students have a high level of applied professional behaviour. As part of the reliability study of the scale, the internal consistency reliability coefficient Cronbach alpha was determined to be 0.95 [18]. In this study, Cronbach alpha was calculated as 0.90.

#### 3.3.3 Instrument of Professional Attitude for Student Nurses (IPASN)

It was developed by Hisar et al. to assess the professional attitudes of nursing students. The scale is a 5‐point Likert scale consisting of 28 items and eight subscales. Its subscales are “contribution to the increase of scientific information load”, “autonomy”, “cooperation”, “collaboration”, “competence and continuous education”, “participation in professional organisations and professional development”, “working in committees”, “community service” and “ethical codes and theory”. As the score on the scale increases, so does the professional attitude (min: 28, max: 140) [19]. The Cronbach's alpha value of the original scale was 0.90 and for this study it was found to be 0.89.

#### 3.3.4 Passion Scale (PS)

Scale developed by Sigmundsson et al. [20] and adapted to Turkish by Taylan et al. [21]. It is a 5‐point Likert scale that measures the level of passion for success. The scale consists of 8 items under a single dimension. The higher the score, the greater the passion for success. There is no reverse coding. The scale has no cut‐off point. The total Cronbach alpha coefficient in the adaptation study was 0.864 and the Cronbach alpha coefficient in this study was 0.851.

#### 3.3.5 Grit‐ S Scale (GS)

The short grit scale, developed by Duckworth and Quinn [6] and adapted into Turkish by Sarıçam, Çelik and Oğuz [22], is a 5‐point Likert type measurement tool used for self‐assessment [6]. The scale consists of eight items and two subscales (consistency of interest and persistence of effort). The Cronbach alpha value of the scale adaptation was 0.82 [22]. In this study, it was found to be 0.812.

### 3.4 Data Collection

Data were collected in the classroom using data collection forms provided by the researchers from students who agreed to participate in the research after being informed about the study. It took an average of 15–20 min to complete the forms.

### 3.5 Ethical Approval

Before starting the research, approval was obtained from the Non‐Interventional Ethics Committee of a university and written permission was obtained from the institution where the research was conducted (Decision no: KAEK‐234). The necessary permissions to use the scales were obtained from the authors. The purpose of the study was explained to the participants. Students were told that they could withdraw from the study at any time and that the study was voluntary.

### 3.6 Data Analysis

Data were analysed using SPSS 23 (Statistical Package of Social Science) with a 95% confidence interval and a significance level of *p* < 0.05. Descriptive statistical methods (frequency, percentage, mean score, standard deviation) were used to evaluate the data. The relationship between students' professional attitudes and behaviours was assessed using multiple linear regression analysis. Student characteristics, PS and GS scale scores were included in the models created for regression analysis. The stepwise method was used to decide whether or not to include variables in the model. *R* ^2^ was calculated for the amount of change in the data defined by the linear regression, Durbin–Watson was calculated to test if the terms were correlated according to the regression model, and Variance Inflation Factor (VIF) and tolerance statistics were calculated to determine if there was multicollinearity in the regression model.**3**.

## 4 Results

### 4.1 IPASN, NSPBS, PS, and GS Total and Subscale Mean Scores

The analysis revealed that the Total IPASN mean score was 85.43 ± 16.22, with the highest subscale score in “Contribution to the increase of scientific information load” (16.64 ± 3.721) and the lowest subscale score in “Working in committees” (7.85 ± 1.565). For the NSPBS, the total mean score was 62.06 ± 7.89, with the highest score observed in “Health care practices” (23.43 ± 7.18) and the lowest in “Reporting” (7.35 ± 1.86). Additionally, the PS score was 28.74 ± 5.82, while the GS total score was 25.64 ± 3.721, with subscale scores for “Consistency of Interest” (12.55 ± 2.003) and “Perseverance of Effort” (13.198 ± 2.037) (Table 1).

**TABLE 1:** Total and subscale mean scores for IPASN, NSPBS, PS and GS.

Scale and sub‐dimensions	Score ranges	Scale scores of nurses
**Total IPASN mean score**	44.00–140.00	85.43 ± 16.22 (48–125)
Contribution to the increase of scientific information load	6.00–30.00	16.64 ± 3.721 (7–30)
Autonomy	3.00–15.00	10.28 ± 2.82 (3–15)
Cooperation	5.00–25.00	11.36 ± 3.157 (3–15)
Competence and continuous education	3.00–15.00	9.93 ± 3.004 (3–15)
Participation in professional organisations and professional development	3.00–15.00	9.41 ± 2.88 (3–15)
Working in committees	2.00–10.00	7.85 ± 1.565 (2–10)
Community service	3.00–15.00	10.17 ± 2.840 (3–15)
Ethical codes and theory	3.00–15.00	9.36 ± 2.882 (3–15)
**Total NSPBS mean score**	27.00–135.00	62.06 ± 7.892 (37–126)
Health care practices	18.00–90.00	23.43 ± 7.183 (18–86)
Activity practice	7.00–35.00	31.28 ± 4.08 (7–35)
Reporting	2.00–10.00	7.35 ± 1.862 (2–10)
**PS**	8.00–40.00	28.74 ± 5.82 (13–40)
**GS**	8.00–40.00	25.64 ± 3.721 (16–39)
Consistency of interest	4.00–20.00	12.55 ± 2.003 (4–20)
Perseverance of effort	4.00–20.00	13.198 ± 2.037 (4–20)

Abbreviations: GS, Grit‐S scale; IPASN, Instrument of Professional Attitude for Student Nurses; NSPBS, Nursing Students Professional Behaviours Scale; PS, Passion Scale.

### 4.2 Socio‐Demographic Characteristics

The average age of the nursing students was 20.93 ± 1.809 (18–26). Most of the students were between the ages of 18–21, comprising 61.2% of the sample. Regarding their academic standing, 31.0% of the students were in their fourth year. The majority of participants were female, accounting for 67.6%. In terms of economic status, 47.3% of the students perceived their income as lower than their expenditure. When considering the reasons for choosing their profession, 47.8% of the students reported choosing the profession due to anxiety about finding a job, while 45.9% chose it voluntarily. As for post‐graduation goals, 58.5% of the students expressed a desire to work as a nurse (Table 2).

**TABLE 2:** Distribution of socio‐demographic characteristics (*n* = 207).

Socio‐demographic characteristics	*n*	%
Age 20.93 ± 1.809 (18–26)		
18–21	134	61.2
22–24	62	35.4
25–27	11	3.5
Class		
II	68	28.1
III	69	19.4
IV	70	31.0
Gender		
Female	140	67.6
Male	67	32.4
Economic status		
Income < expenditure	98	47.3
Income = expenditure	94	45.4
Income > expenditure	15	7.2
Reason for choosing the profession		
Choosing a profession voluntarily	95	45.9
Choosing a profession due to family pressure	13	6.3
Choosing a profession due to anxiety about finding a job	99	47.8
Post graduation goal		
Working as a nurse	121	58.5
Make an academic career	67	32.4
Working in a different industry	19	9.1

### 4.3 Correlation of IPASN and NSPBS Total and Subscale Scores With PS and GS Total and Subscale Scores

The study found that there was a positive correlation between students' passion and grit scores and their professional attitude and behaviour scores (Table 3). It was found that the correlational relationship was at a low level between the passion score and the professional attitude scores and the health care practices subscale of the professional behaviour score, and at a medium level between the NSPBS total score and the activity practice and reporting scores. There was a low‐level relationship between the subscale and total scores of the GS and the cooperation and ethical codes and theory subscales of the professional attitude scale, a high‐level relationship with the contribution to increasing scientific knowledge load scores, and a medium‐level relationship with the professional attitude scale total score and other subscale scores. A moderate relationship was found with the total and subscale scores of the perseverance scale, the total score and the health care practices subscale score of the professional behaviour scale, and a low relationship with the other subscale scores (Table 3).

**TABLE 3:** Correlation of IPASN and NSPBS to total and subscale scores with PS and GS total and subscale scores.

	PS	GS		
		Total GS	Consistency of interest	Perseverance of effort
**IPASN**				
Total IPASN				
*r*	0.255	0.526	0.489	0.543
*p*	0.000	0.000	0.000	0.000
Contribution to the increase of scientific information load				
*r*	0.230	0.802	0.848	0.790
*p*	0.001	0.000	0.000	0.000
Autonomy				
*r*	0.209	0.472	0.408	0.481
*p*	0.002	0.000	0.000	0.000
Cooperation				
*r*	0.244	0.171	0.155	0.140
*p*	0.000	0.014	0.025	0.044
Competence and continuous education				
*r*	0.246	0.429	0.328	0.375
*p*	0.000	0.000	0.000	0.000
Participation in professional organisations and professional development				
*r*	0.256	0.252	0.209	0.289
*p*	0.000	0.000	0.007	0.000
Working in committees				
*r*	0.174	0.336	0.302	0.358
*p*	0.012	0.000	0.000	0.000
Community service				
*r*	0.188	0.505	0.395	0.463
*p*	0.007	0.000	0.000	0.000
Ethical codes and theory				
*r*	0.150	0.260	0.170	0.247
*p*	0.031	0.000	0.015	0.000
**NSPBS**				
Total NSPBS				
*r*	0.344	0.381	0.365	0.342
*p*	0.000	0.000	0.000	0.000
Health care practices				
*r*	0.242	0.319	0.305	0.301
*p*	0.000	0.000	0.000	0.000
Activity practice				
*r*	0.557	0.189	0.223	0.146
*p*	0.000	0.006	0.001	0.035
Reporting				
*r*	0.388	0.197	0.203	0.192
*p*	0.000	0.005	0.001	0.004

Abbreviations: GS, Grit‐S scale; IPASN, Instrument of Professional Attitude for Student Nurses; NSPBS, Nursing Students Professional Behaviours Scale; PS, Passion Scale.

### 4.4 Students' Professional Attitudes and Behaviours Using Stepwise Multiple Linear Regression

To ensure the validity of our linear regression models, we thoroughly assessed the fundamental assumptions. Linearity was tested using scatter plots comparing residuals with predicted values, confirming a linear relationship. Homoscedasticity was checked by examining residual plots, and constant variance was observed. Independence of errors was evaluated using the Durbin–Watson statistic, with values of 1.557 for professional attitude models and 1.511 for professional behaviour models, indicating no significant autocorrelation. Multicollinearity was assessed using the Variance Inflation Factor (VIF), and all variables had VIF values below 2 (ranging from 1.003 to 1.091), indicating the absence of multicollinearity issues.

In the initial regression analysis, we included a comprehensive set of variables to explore the factors influencing professional attitudes and behaviours among nursing students. These variables encompassed socio‐demographic characteristics (age, gender, year of study, economic status, and post‐graduation goals) as well as factors related to professional attitudes. During the stepwise regression process, variables that did not demonstrate a statistically significant contribution to the model were systematically excluded. Specifically, some socio‐demographic variables were removed during this process due to their lack of significant association with the dependent variable. The final model retained only those variables that significantly contributed to explaining the variance in professional attitudes and behaviours.

When students' professional attitudes were examined, the best model was IPASN Total, in step 3 (Table 4); when students' professional behaviours were examined, the best model was formed in step 4 of the NSPBS Total (Table 5). The Durbin–Watson value ensured model validity in all models of professional attitudes (Total IPASN D = 1.557) and professional behaviour (Total NSPBS D = 1.511).

**TABLE 4:** Stepwise multiple linear regression model of professional attitude.

Model	*R*	*R* ^2^	Adjusted *R* ^2^	SE of estimation	*F* change	Sig. *F* change	Durbin–Watson
1	0.637	0.406	0.403	26.01815	138.816	0.000	1.557
2	0.648	0.420	0.414	25.78652	4.663	0.000	
3	0.656	0.431	0.422	25.59917	3.968	0.011	

Coefficients of model 3							
Variables	*B*	SE	*β*	*t*	Sig.	Tolerance	VIF
(Constant)	12.428	13.824		0.899	0.370		
GS	5.559	4.937	0.615	11.193	0.000	0.939	1.065
II. grade students	−11.118	4.934	−0.120	−2.254	0.025	0.997	1.003
PS	0.636	0.319	0.109	1.992	0.048	0.933	1.066

Abbreviations: GS, Grit‐S scale; PS, Passion Scale.

**TABLE 5:** Stepwise multiple linear regression model of professional behaviour.

Model	*R*	*R* ^2^	Adjusted *R* ^2^	SE of estimation	*F* change	Sig. *F* change	Durbin–Watson
1	0.532	0.283	0.279	15.14882	80.728	0.000	1.511
2	0.656	0.430	0.425	13.53356	52.855	0.000	
3	0.690	0.476	0.468	13.01577	17.554	0.000	
4	0.702	0.493	0.483	12.83275	6.832	0.010	

Coefficients of model 4							
Variables	*B*	SE	*β*	*t*	Sig.	Tolerance	VIF
(Constant)	18.907	7.008		2.698	0.008		
II. Grade students	−18.926	1.922	−0.499	−9.847	0.000	0.930	1.075
GS (grade students)	1.612	0.249	0.336	6.473	0.000	0.917	1.091
PS (professional skill)	0.629	0.160	0.205	3.926	0.000	0.966	1.035
Choosing nursing voluntarily	4.812	1.841	0.133	2.614	0.010	0.976	1.024

Abbreviations: GS, Grit‐S scale; PS, Passion Scale.

It was found that being a second‐year student reduced the IPASN total score by 0.120 points (Table 4) and the NSPBS total score by 0.499 points (Table 5). It was found that choosing nursing voluntarily increased the NSPBS total score by 0.139 points (Table 5). It was determined that a one‐point increase in the GS standard deviation increased the standard deviation of total IPASN scores by 0.615 points (Table 4) and the standard deviation of total NSPBS scores by 0.336 points (Table 5).

It was determined that a one‐point increase in the PS standard deviation increased the standard deviations of total IPASN scores by 0.109 points (Table 4) and total NSPBS scores by 0.205 points (Table 5).

## 5 Discussion

This study aims to examine the relationship between passion and grit and professional attitudes and behaviours in nursing students based on grit theory. Understanding the role of passion and grit in students' professional attitudes and behaviours provides evidence that characteristics such as passion and grit are important for nursing programmes and educators to support students' professional development.

Looking at the average of the total Grit and Passion scale scores of the nursing students in the study, it can be said that the students have a medium level of Grit and Passion. Although there are a limited number of studies in the literature evaluating the grit levels of nursing students [9, 16, 23] no study has been found to evaluate their passion levels. For most students, their time at university is an important process in which they develop by immersing themselves in different activities in early adulthood. They develop their sense of self through involvement in different activities that they can be passionate about. They develop their sense of self through involvement in different activities that they can be passionate about. During their time at university, students are likely to have a variety of personal and professional goals. Progress towards these goals requires patience and consistency—in other words, grit [3]. Passion and grit have been evaluated together in recent studies [2, 3, 24]. In the field of nursing, much research is needed to assess passion and grit.

Previous studies have also shown that the grit level of nursing students is moderate to high [9, 16, 23]. In addition, the scores obtained for the subscales of grit in Kang and Koinin's study showed that continuity of effort was higher than continuity of interest, which is consistent with our study. Continuity of interest refers to the passion to maintain a goal or area of interest over a relatively long period of time. Resistance to effort means patience and includes being patient, especially in overcoming obstacles and difficulties [17, 25]. In other words, grit expresses a commitment and passionate resistance that allows us to overcome challenges and exert sustained effort to achieve our goals. The third and fourth‐year students in the study may lack passion, or in other words continuous interest, due to the long and challenging academic process. It is necessary to consider different educational aspects and to look for concrete measures so that students can maintain their interest and continuity throughout their further studies.

The study found that the professional attitudes and behaviours of nursing students were at a moderate level. It was found that nursing students' professional attitudes and behaviours varied when reviewing the literature. In some studies, the professional attitudes and behaviours of nursing students are at a medium level or above [13, 21, 26], while in other studies they are quite low [15, 27]. Identifying the factors that influence professional attitudes and behaviours in nursing students is important for developing strategies to improve positive attitudes and behaviours.

As grit theory postulates, grit is considered to be strong willpower that keeps people committed to long‐term goals in the face of difficulty. In terms of an individual's long‐term success or ‘resilience’ in their career, this quality plays a particularly important role [5]. In support of grit theory, our study results found a positive relationship between grit and professional attitudes and behaviours among nursing students. According to the results of the regression analysis, it was found that as the grit and passion levels of the nursing students increased, so did their professional attitudes and behaviours. No study was found in the literature that examined the relationship between grit and passion and the professional attitudes and behaviours of nursing students. Grit, and the passion that is an integral part of it, is a new paradigm in nursing. As a result, there is limited research on the impact of grit and passion in nursing education. In these studies, grit was found to be strongly related to the academic and clinical performance of nursing students [9, 16, 23]. By understanding and strengthening the grit and passion of nursing students, educators and institutions can better support their success in academic and clinical settings and their professional attitudes and behaviours.

In the study, being a second‐year student had a negative impact on the professional attitudes and behaviours of nursing students. Evidence from the literature suggests that the relationship between the year level of nursing students and their professional attitudes and behaviours is variable. In some studies, professional attitudes and behaviours increased as grade level increased [28, 29], while in others, professional attitudes and behaviours decreased as grade level increased [13, 30]. The differences between nursing students' grade levels and professional attitudes and behaviours may be due to various factors, such as students' personal experiences, the structure of educational programmes, and differences in the learning process. More research is needed to understand this relationship.

In the study, the voluntary choice of nursing had a positive effect on the professional attitudes and behaviour of nursing students. Similarly, two studies investigating factors influencing professional behaviour found that the professional behaviour scores of students who voluntarily chose nursing were high [13, 31]. Choosing the profession voluntarily may have enabled students to be more passionate and steadfast in their academic and clinical practice, and to adopt professional attitudes and behaviours more easily.

The study has several limitations. The cross‐sectional design of the study limits causal inferences. Although the scales used in the study are popular and comprehensive the levels of grit and passion are limited to the definitions of these scales. The strength of the study is that it is the first study to examine the relationship between grit and passion and professional attitudes and behaviours in nursing students.

## 6 Conclusion

As predicted in this study based on grit theory, a positive relationship was found between nursing students' passion and grit levels and their professional attitudes and behaviours. As nursing students' grit and passion increased, so did their professional attitudes and behaviours. The results of this study highlight the importance of personal characteristics such as passion and grit in influencing nursing students' professional attitudes and behaviours. In this regard, educators and nursing programmes can develop various strategies to enhance students' passion and grit. Students can be provided with an encouraging and supportive environment to help them cope with the challenges they may experience throughout their careers. It is recommended that studies are undertaken to provide evidence on the effectiveness of different interventions to increase grit in nursing students.

## 7 Relevance of Clınıcal Practıce

The findings of this research highlight the significant role that grit and passion play in shaping nursing students' professional attitudes and behaviours. These results suggest that nursing education programmes should incorporate strategies aimed at fostering and enhancing grit and passion in students. Specifically, educators and institutions should create a supportive learning environment that motivates students in both clinical and academic settings, helping them develop resilience in the face of challenges. Integrating practices that promote grit and passion into nursing curricula may lead to improved professional performance and higher quality patient care in the long run. Moreover, these findings offer a foundation for future research to explore effective interventions that can further strengthen grit and passion among nursing students, ultimately contributing to their professional growth and success.

## Author Contributions

**Seçil Taylan:** conceptualization, methodology, data collection, writing – original draft, supervision. **İlknur Özkan:** data analysis, writing – review and editing, visualization, correspondence, project administration. Both authors read and approved the final manuscript.

## Conflicts of Interest

The authors declare no conflicts of interest.

## Data Availability

The data that support the findings of this study are available on request from the corresponding author. The data are not publicly available due to privacy or ethical restrictions.
